# Birds of a Feather: Neanderthal Exploitation of Raptors and Corvids

**DOI:** 10.1371/journal.pone.0045927

**Published:** 2012-09-17

**Authors:** Clive Finlayson, Kimberly Brown, Ruth Blasco, Jordi Rosell, Juan José Negro, Gary R. Bortolotti, Geraldine Finlayson, Antonio Sánchez Marco, Francisco Giles Pacheco, Joaquín Rodríguez Vidal, José S. Carrión, Darren A. Fa, José M. Rodríguez Llanes

**Affiliations:** 1 The Gibraltar Museum, Gibraltar; 2 Department of Social Sciences, University of Toronto, Toronto, Canada; 3 Àrea de Prehistòria, Universitat Rovira i Virgili (URV), Tarragona, Spain; 4 IPHES, Institut Català de Paleoecologia Humana i Evolució Social, C/Marcel·lí Domingo s/n (Edifici W3), Campus Sescelades, Tarragona, Spain; 5 Estación Biológica de Doñana (CSIC), Avda,Americo Vespucio s/n, Sevilla, Spain; 6 Department of Biology, University of Saskatchewan, Saskatoon, Canada; 7 Area of Neogene and Quaternary Faunas, Institut Català de Paleontologia, Campus de la UAB, Cerdanyola del Vallès, Spain; 8 Depto. Geodinámica y Paleontología, Facultad de Ciencias Experimentales, Campus del Carmen, Universidad de Huelva, Huelva, Spain; 9 Department of Plant Biology, University of Murcia Campus de Espinardo, Murcia, Spain; 10 CRED - Centre for Research on the Epidemiology of Disasters Institute Health and Society, Université Catholique de Louvain 30, Brussels, Belgium; University of Oxford, United Kingdom

## Abstract

The hypothesis that Neanderthals exploited birds for the use of their feathers or claws as personal ornaments in symbolic behaviour is revolutionary as it assigns unprecedented cognitive abilities to these hominins. This inference, however, is based on modest faunal samples and thus may not represent a regular or systematic behaviour. Here we address this issue by looking for evidence of such behaviour across a large temporal and geographical framework. Our analyses try to answer four main questions: 1) does a Neanderthal to raptor-corvid connection exist at a large scale, thus avoiding associations that might be regarded as local in space or time?; 2) did Middle (associated with Neanderthals) and Upper Palaeolithic (associated with modern humans) sites contain a greater range of these species than Late Pleistocene paleontological sites?; 3) is there a taphonomic association between Neanderthals and corvids-raptors at Middle Palaeolithic sites on Gibraltar, specifically Gorham's, Vanguard and Ibex Caves? and; 4) was the extraction of wing feathers a local phenomenon exclusive to the Neanderthals at these sites or was it a geographically wider phenomenon?. We compiled a database of 1699 Pleistocene Palearctic sites based on fossil bird sites. We also compiled a taphonomical database from the Middle Palaeolithic assemblages of Gibraltar. We establish a clear, previously unknown and widespread, association between Neanderthals, raptors and corvids. We show that the association involved the direct intervention of Neanderthals on the bones of these birds, which we interpret as evidence of extraction of large flight feathers. The large number of bones, the variety of species processed and the different temporal periods when the behaviour is observed, indicate that this was a systematic, geographically and temporally broad, activity that the Neanderthals undertook. Our results, providing clear evidence that Neanderthal cognitive capacities were comparable to those of Modern Humans, constitute a major advance in the study of human evolution.

## Introduction

The regular and systematic exploitation of flying birds for food is considered to be a hallmark of behavioural modernity, exclusive to anatomically modern *Homo sapiens* (Modern Humans) after 50 thousand years ago (kya) [1], [2]. The prevailing paradigm among Palaeolithic archaeologists today is still one which regards flying birds to have been difficult prey to capture and beyond the capabilities of all hominins prior to 50 kya and non-modern hominins (including the Neanderthals) even after the 50 kya threshold [1], [2]. The corollary, which has been applied to the Neanderthals for the period after 50 kya, is that they only targeted birds once easier prey (presumed to be energetically less costly to obtain than birds) were exhausted [3], [4]. Even when evidence that the Neanderthals took prey commonly regarded as difficult has been presented [5], the argument that these are examples of opportunistic and unsystematic captures has been used in response [6]. These interpretations have been contested from an ecological perspective which suggests that Neanderthals were equally versatile omnivorous hunter-gatherers [7], who even included marine mammals in their diet when available [5]. Recently, evidence has been accumulating that strongly suggests that Neanderthals regularly exploited birds as part of a varied diet within coastal Mediterranean regions [8], [9], [10].

However, the hypothesis that Neanderthals exploited birds for the use of their feathers or claws as personal ornaments in symbolic behaviour [11], [12] is revolutionary as it assigns unprecedented cognitive abilities to these hominins. Specifically, raptors (Orders Accipitriformes and Falconiformes) and corvids (Family Corvidae in the Order Passeriformes) were among the bird taxa found associated with Neanderthals at Riparo Fumane, Italy [11] and, Combe-Grenal and Les Fieux, France [12]. The suggestion that Neanderthals exploited birds for ornamental purposes has added a further and important dimension to the debate, that of their cognitive capacities. This hypothesis has, however, been put forward on the basis of very small samples and is thus open to the criticism that it does not represent regular or systematic behaviour.

To assess the existence of universal patterns of early use of feathers for ornamental and symbolic purposes, here we examine the relationship between Modern Humans, Neanderthals, raptors and corvids across a broad temporal and geographical framework: the Palearctic Region in the Middle and Late Pleistocene. These taxa of birds are chosen for the present study because (a) they are frequently present in sites occupied by hominins; (b) they represent taxa that are not typically consumed by hominins; and (c) they are carnivores that often scavenge the corpses of medium and large mammals, so that they were likely to frequently come into close contact with humans. They may have, in all likelihood, also been regular scavengers around Palaeolithic camp sites [13], as they are today in urban areas and garbage dumps in many parts of the world [14]. Corvids are abundant species in many Eurasian landscapes while raptors - apex predators - tend to be scarcer. Both groups include rock dwelling species that would be naturally expected to accumulate close to nesting sites but there are no known taphonomic processes that would concentrate the remains from these taxa any more than other rock-dwelling birds. In any case our findings also include species that are tree nesters as well. For these reasons the palaeontological sites would seem to reflect natural accumulation rates.

From a multi-scale approach, we show that strong positive relationships exist between Neanderthal-raptor and corvid. On the other hand, we confirm, using taphonomic data from three sites in Gibraltar (Gorham's, Vanguard and Ibex Caves), that the relationship involves active processing of raptors and corvids by Neanderthals for the purpose of wing feather removal. The temporal and geographical extent of the connection, along with the direct taphonomic evidence, establishes that Neanderthals systematically targeted these birds for purposes other than food.

## Analysis

In order to solve the problems related to small samples, we address four specific questions regarding the Neanderthal-raptor and corvid relationship. First, we asked whether a connection existed at the largest possible scale, thus avoiding associations that might be regarded as local in space or time. To do this we looked at sites covering the entire Pleistocene and the whole of the Palearctic Region. Second, we asked the question did Middle (associated with Neanderthals) and Upper Palaeolithic (associated with Modern Humans) sites contain a greater range of these species than paleontological sites? This was done to establish whether the observed associations were related to hominin activity. Third, to try and determine the nature of the association we undertook a taphonomic examination of the bones of these birds from the site with the most species of the 1699 sites in our database. Finally, we asked whether the observed behaviour -the extraction of wing feathers - was a local phenomenon exclusive to the Neanderthals at the site studied or whether it was, instead, a geographically wider phenomenon.

To answer the first question, whether there was a broad temporal and geographical relationship between hominins, raptors and corvids, we compiled a database of 1699 Pleistocene Palearctic sites, based on fossil bird sites have been catalogued by Tyrberg [15], [16]. This database included all raptor and corvid species as well as corresponding archaeological and paleontological attribution (Table S1). Table 1 summarises the results of the analysis of 1699 Palearctic Pleistocene sites. These results are striking because they show a clear over-representation of bird species with dark remiges (wing feathers) in Palaeolithic sites when compared to paleontological sites with no human presence (X^2^_1_ = 8.667, p = 0.003, Text S1). It is particularly significant that the relationship holds for two unrelated lineages of birds (families Accipitridae and Corvidae). The relationship was also found to be stronger in the Middle (typical of Neanderthals) than the Upper Palaeolithic (typical of Modern Humans; X^2^_1_ = 7.278, p = 0.007, Text S1). In contrast, we found no statistically significant differences in the sizes of the species present in Palaeolithic versus paleontological sites (from Table 1) which indicates that they were not being chosen for large size. Table 1 also shows a clear over-representation of scavenging birds in Palaeolithic sites when compared to paleontological sites with no human presence (X^2^_1_ = 11.026, p<0.001). The relationship also holds across unrelated lineages with similar scavenging habits. In addition, we found several examples of species that were overrepresented in Middle Palaeolithic sites when compared with Upper Palaeolithic ones; we found no cases in which there was over-representation in Upper over Middle Palaeolithic sites. In contrast, a range of raptors and corvids that rarely, if at all, scavenge at carcasses were found to occur in Palaeolithic sites at similar frequency to paleontological sites. Thus we conclude that there is a positive association between humans and scavenging birds, especially marked for some species in the Middle Palaeolithic. A second group of birds also appeared strongly associated with Palaeolithic, especially Middle, sites. These were two species of *Pyrrhocorax* choughs (Corvidae) and two *Falco* kestrels (Falconidae). These birds are not scavengers but are all cliff nesters and three of the four are colonial. Cave-dwelling Neanderthals would have easy access to, or at least regular contact with, these bird species.

**Table 1:** The association of raptors and corvids with Paleolithic humans across the Palearctic.

	Species (vernacular)	Species (scientific)	Behavioural Status	Size Class	Remige color	Over-repr. in PS	Over-repr. in MPS
**Scavengers**	Black Vulture	*Aegypius monachus*	scavenger type 1	6	d	yes***	yes***
	Griffon Vulture**	*Gyps fulvus*	scavenger type 1	6	d	yes***	yes*
	Bearded Vulture*	*Gypaetus barbatus*	scavenger type 1	5	d	yes***	no
	Golden Eagle*	*Aquila chrysaetos*	scavenger type 2	5	d	yes***	no
	Raven*	*Corvus corax*	scavenger type 2	4	d	yes***	no
	White-tailed Eagle*	*Haliaeetus albicilla*	scavenger type 2	5	d	yes**	yes*
	Carrion Crow	*Corvus corone*	scavenger type 2	3	d	yes**	no
	Magpie	*Pica pica*	scavenger type 2	3	m	yes**	no
	Jackdaw	*Corvus monedula*	scavenger type 2	3	d	yes*	yes*
	Rook	*Corvus frugilegus*	scavenger type 2	3	d	yes*	yes*
	Rough-legged Buzzard	*Buteo lagopus*	scavenger type 2	4	i	yes*	no
	Egyptian Vulture*	*Neophron percnopterus*	scavenger type 2	5	d	possible	no
	Black Kite	*Milvus migrans*	scavenger type 2	3	i	possible	no
	Red Kite	*Milvus milvus*	scavenger type 2	4	m	possible	no
	Tawny Eagle	*Aquila rapax*	scavenger type 2	5	i	no	no
	Imperial Eagle	*Aquila heliaca*	scavenger type 2	5	d	no	no
	Spotted Eagle	*Aquila clanga*	scavenger type 2	5	d	no	no
	Common Buzzard	*Buteo buteo*	scavenger type 2	4	i	no	no
**Non-scavenging cliff nesters**	Red-billed Chough	*Pyrrhocorax pyrrhocorax*	cliff colonial	3	d	yes***	yes***
	Lesser Kestrel	*Falco naumanni*	cliff colonial	2	i	yes***	yes**
	Kestrel	*Falco tinnunculus*	cliff solitary	3	i	yes***	yes**
	Alpine Chough	*Pyrrhocorax graculus*	cliff colonial	3	d	yes***	yes*
	Red-footed Falcon	*Falco vespertinus*	partly cliff colonial	3	i	yes**	no
	Gyr Falcon	*Falco rusticolus*	cliff solitary	4	i	yes*	no
	Eleonora's Falcon	*Falco eleonorae*	cliff colonial	3	i	no	no
	Peregrine Falcon	*Falco peregrinus*	cliff solitary	4	i	no	no
	Bonelli's Eagle	*Aquila fasciata*	cliff solitary	5	i	no	no
**Other species**	Eurasian Hobby	*Falco subbuteo*	none	3	i	yes*	no
	Honey Buzzard	*Pernis apivorus*	none	3	i	no	no
	Short-toed Eagle	*Circaetus gallicus*	none	5	i	no	no
	Marsh Harrier	*Circus aeruginosus*	none	4	i	no	no
	Hen Harrier	*Circus cyaneus*	none	3	i	no	no
	Pallid Harrier	*Circus macrourus*	none	3	i	no	no
	Montagu's Harrier	*Circus pygargus*	none	3	i	no	no
	Northern Goshawk	*Accipiter gentilis*	none	3	i	no	no
	Eurasian Sparrowhawk	*Accipiter nisus*	none	4	i	no	no
	Long-legged Buzzard	*Buteo rufinus*	none	4	i	no	no
	Lesser Spotted Eagle	*Aquila pomarina*	none	5	d	no	no
	Booted Eagle	*Hieraaetus pennatus*	none	4	i	no	no
	Osprey	*Pandion haliaetus*	none	5	i	no	no
	Merlin	*Falco columbarius*	none	3	i	no	no
	Saker Falcon	*Falco cherrug*	none	4	i	no	no
	Jay	*Garrulus glandarius*	none	3	m	no	no
	Nutcracker	*Nucifraga caryocatactes*	none	3	d	no	no

The table is divided into three sections, the first covering scavenging birds, the second non-scavenging cliff-nesting birds and the third covering the remaining species. Scavengers are separated into type 1 (obligate) and type 2 (facultative, ranging from frequent to occasional). Scavengers that are also cliff nesters are assigned an * if they are solitary nesters and ** if they are colonial. Each species is allocated to a size class according to the following scale: 1 - all individuals <100 g; 2 - some individuals <100 g and others between 100 and 1 kg; 3 – all individuals between 100 and 1 kg; 4 – some individuals between 100 g-1 kg and others between 1–10 kg; 5 all individuals between 1 and 10 kg; and 6 some individuals between 1–10 kg and others >10 kg. Species which are overrepresented in Palaeolithic sites (Middle and Upper) compared to paleontological sites, tested by chi-square (Text S1), are indicated by a “yes”. Species that are overrepresented in Middle over Upper Palaeolithic sites are similarly indicated. Degree of significance: *** p<0.001; **p<0.01; *p<0.05. Cases of possible overrepresentation in Palaeolithic sites but with sample sizes that are too small to provide definitive evidence are indicated as “possible”. Remige feather colour: d = dark; i = intermediate - this includes birds with light brown or more often spotting or barred patterns so have some white and some dark per feather; and m = mix where some feathers are white (e.g. primaries) and some are black (e.g. secondaries). Over-repr. = over-represented; PS = Palaeolithic sites; MPS = Middle Palaeolithic sites. Statistical analyses are provided in Tables S6 and Text S1.

To answer the second question, whether Middle and Upper Palaeolithic sites contained a greater range of raptor and corvid species than paleontological sites, we listed how many of the scavengers (including the three categorised as possible in Table 1), choughs and kestrels were present in each of the 1699 sites. The suite of species numbered 18 (Table S1). Our results showed that Middle and Upper Palaeolithic sites did contain more raptor and corvid species than paleontological sites: 47 Middle Palaeolithic and 55 Upper Palaeolithic sites had six or more species while only 31 palaeontological sites did so; 136 Middle Palaeolithic and 260 Upper Palaeolithic sites had between 1 and 5 species while 355 paleontological sites had this number; finally, only 59 Middle Palaeolithic and 210 Upper Palaeolithic sites had none of the species while 607 paleontological sites fell in this category. The results were highly statistically significant (X^2^_4_ = 171.298, p<0.0001). Comparing Middle with Upper Palaeolithic sites also revealed an excess of sites with over six species in the Middle Palaeolithic (X^2^_2_ = 22.92, p<0.0001). So hominin sites tended to be associated with a large element of the suite of 18 species identified, Middle Palaeolithic sites more so than Upper Palaeolithic ones. Apart from the complex taphonomic histories of the archaeological sites, these results indicate a striking association between hominins, especially Neanderthals, and a suite of scavenging and colonial cliff nesting raptors and corvids which characteristically have dark remiges. The fact that three different phylogenetic lineages (raptors, falcons and corvids), with similar ecologies [17] were represented, while others in the same lineage but with different ecologies were not, strongly indicates that the relationship had a strong ecological signal.

We attempted to answer the third question, regarding the nature of the observed association, by examining the bones of raptors and corvids from Gorham's Cave, Gibraltar, which was the site with the most species (16 of 18) represented in our database of 1699 sites (Table S1). We also examined, for comparison, bones from two other Middle Palaeolithic sites on Gibraltar: Vanguard Cave (with 7 species) and Ibex Cave (with 8).

**Figure 1:**
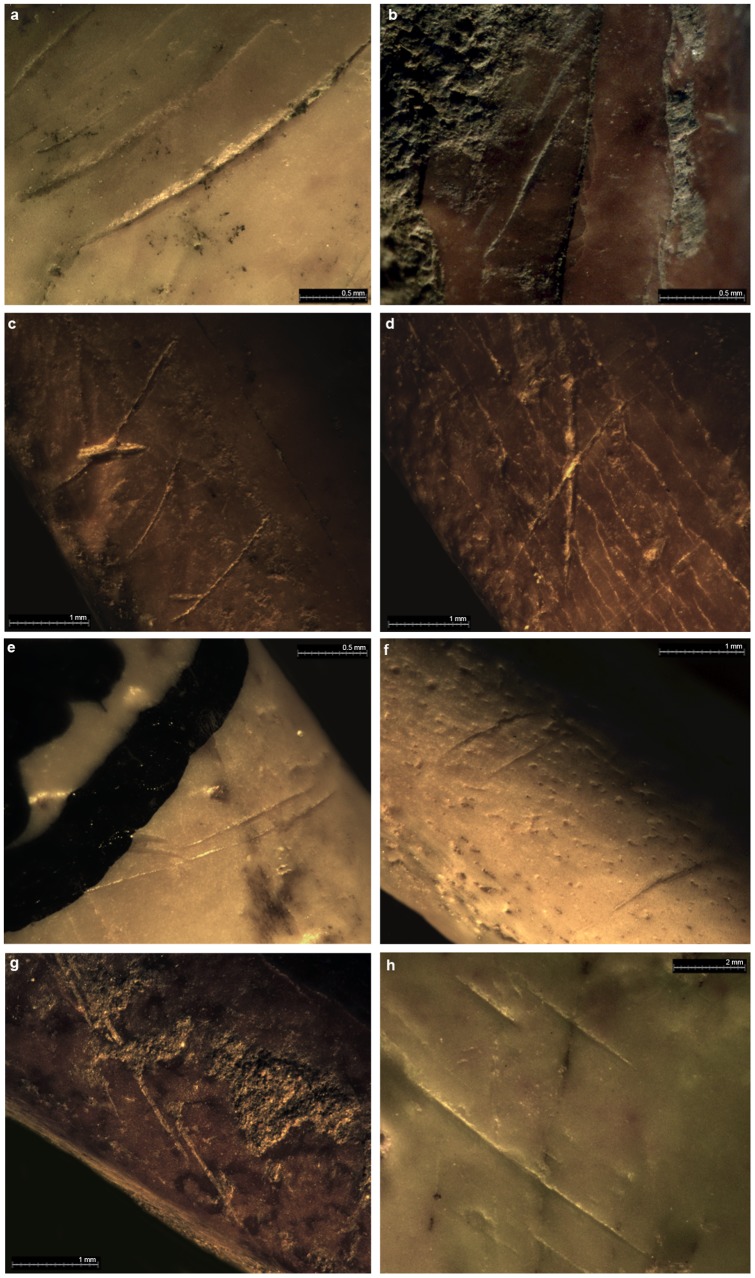
Examples of cut-marks from Gibraltar sites. a) distal diaphysis of *Pyrrhocorax pyrrhocorax* humerus (Gor'96 No. 87); b) proximal diaphysis of *Pyrrhocorax pyrrhocorax* humerus; c) proximal diaphysis of *Pyrrhocorax pyrrhocorax* humerus (GOR'96 NO. 299); d) distal diaphysis of *Milvus milvus* radius (GOR'00/B8/NIV/205); e) middle shaft of *Pyrrhocorax pyrrhocorax* tarsometatarsus (Ibex 94 No. 24); f) middle shaft of *Pyrrhocorax pyrrhocorax* femur (Ibex 94 No. 166); g) proximal diaphysis of *Pyrrhocorax graculus* ulna (GOR'00/B5/NIV/57); h) distal diaphysis of *Gyps fulvus* ulna (Van 96 No. 209A).

We examined a total of 604 skeletal elements (NISP) from 21 species of raptors, falcons and corvids (Table 2, Text S2). Notably, they included 7 species of our suite of 18 identified for the whole Palearctic: Golden Eagle *Aquila chrysaetos*, Griffon Vulture *Gyps fulvus*, Black Kite *Milvus migrans*, Red Kite *M. milvus*, Carrion Crow *C. corone*, Red-billed Chough *Pyrrhocorax pyrrhocorax* and Alpine Chough *P. graculus*. These NISP were distributed into 486 from Gorham's Cave, 91 from Vanguard Cave and 27 from Ibex Cave. 33 of the 604 elements (5.46%) showed cut-marks made by Neanderthal stone tools (Figure 1; Table S2 and Table S3); 18 (2.98%) showed bone breakage in fresh state; 3 (0.49%) had been burnt; and one had human tooth imprints. In addition, 9 of 201 ulnae and humeri (4.48%) showed evidence of over-extension (arrachement and peeling). The skeletal elements represented a minimum number (MNI) of 124 individuals. Of these, at least 18 individuals, of the 7 species listed above, showed evidence of direct Neanderthal action on them. The nature of the observed evidence of such action resembled closely that observed in the small Riparo Fumane sample that was interpreted as evidence of feather removal [11]. In contrast, modifications by other agents, such as carnivores or rodents, were negligible. Only 2.3% of all the elements showed marks by carnivore gnawing; 0.5% showed marks by rodent gnawing; and 0.66% showed damage due to digestive action by birds of prey.

**Table 2:** NISP, MNE, MNI and anthropogenic damage on bird remains from Gibraltar sites.

	Gorham's Cave							Vanguard Cave						Ibex Cave			
	NISP	NME	NMI	Cm	Oext	Bur	BnBr	NISP	NME	NMI	Cm	Oext	HTm	NISP	NME	NMI	Cm
*Accipiter gentilis*	2	2	1					2	2	1							
*Accipiter nisus*	11	11	2					6	6	1			1				
*Accipiter* sp.								2	2	1							
*Aquila chrysaetos*	5	5	2	1			1										
*Aquila* sp.	3	3	1											1	1	1	
*Aquila sp./haliaeetus* sp.								1	1	1							
*Buteo buteo*	3	3	1														
*Buteo sp.*								1	1	1							
*Circus cyaneus*								1	1	1							
*Corvus corax*	1	1	1					2	2	1				3	3	1	
*Corvus corone*	9	9	4											2	2	1	
*Corvus corone/frugilegus*	7	7	3														
*Corvus monedula*	58	58	8					16	16	3							
*Falco naumanni*	28	28	5					6	6	3							
*Falco peregrinus*	4	4	2														
*Falco subbuteo*	1	1	1														
*Falco tinnunculus*	34	34	4					4	4	1							
*Falco* sp.								2	2	1							
*Falco* sp./*accipiter* sp.								1	1	1							
*Gyps barbatus*														1	1	1	
*Gyps fulvus*	4	4	1					16	16	3	2	1					
*Gyps melitensis/fulvus*	14	14	2	1													
*Gyps* sp.								1	1	1							
*Gyps/aegypius*	1	1	1					3	3	2							
*Haliaeetus albicilla*								1	1	1							
*Hieraaetus fasciatus*								1	1	1							
*Milvus migrans*	1	1	1	1													
*Milvus milvus*	22	22	5	4	1		2										
*Milvus* sp.	8	8	3					1	1	1							
*Pica pica*	9	9	2														
*Pyrrhocorax graculus*	73	73	11	9	2	2	2	3	3	1							
*Pyrrhocorax pyrrhocorax*	180	178	17	10	5	1	11	17	17	5	1			20	20	3	4
*Pyrrhocorax* sp.	7	7	4				1	1	1	1							
Unident. Bird of prey	1	1	1					3	3	2							
Total	486	484	83	26	8	3	17	91	91	34	3	1	1	27	27	7	4

Cm: cut-marks; Oext: over-extending; Bur: burning; BnBr: fresh bone breakage; HTm: human tooth-marks.

The sample examined showed a clear bias of wing bones over other skeletal elements (Goodness of Fit, G_2_ = 985.4379, p<0.0001). Thus, 337 of the 604 (55.7%) bones were wing bones, compared with 184 leg bones (30.46%) and only 83 (13.74%) from the axial skeleton (Text S3). The over-abundance of wing elements has been a long-standing issue in avian Palaeozoology with some discussion in both the paleontological [18] and in the zooarchaeological literature [19]. Both cultural and post-depositional hypotheses have been proposed to explain this pattern. One of the main explanations for this phenomenon has been the differential survival of avian elements due to questions of bone density. Wing bones may be more likely to survive because they are denser than other skeletal elements, and therefore less likely to be crushed or fragmented. However, bone strength varies significantly among bird species as a result of differential pneumatization, feeding, functional anatomy or type of locomotion [20]. In addition to this, bone density is a complex attribute, whose data are not available for most kinds of birds. Taking into account these limiting factors, a bivariant test between maximum bone density of several skeletal elements and the main represented species (*Pyrrhocorax pyrrhocorax*) was calculated (Table S4). This correlation was only applied to the Gorham's Cave sample because it involves the highest number of bones. On this basis, no differential destruction based on bone density was detected at this site (<0.5) and therefore, fossil-diagenetic processes do not seem to explain the disappearance of some skeletal elements in the analyzed assemblages. From this perspective, several authors state that the abundance of bird wing elements may be a consequence of human activities, such as scavenging, use of feathers, differential transport, processing and consumption [18], [19], [21], [22], [23]. Ethnographic evidence supports this archaeological pattern and has been used by some scholars to formulate predictions for the cultural explanations [21]. The results from the Gibraltar sample are striking because, given the number of NISP, MNI, species and bias towards wing elements, they reveal that the processing of bird bones by Neanderthals was not random and accidental but a regular behavioural activity. This activity was clearly related to the extraction of the largest, most durable, and arguably most visually striking, elements of a bird's plumage. Our conclusion that this was a systematic behaviour is strengthened by the fact that we found evidence for the practice in three caves and different stratigraphic levels in a single cave (Gorham's). Additionally, these levels covered a large part of Marine Isotope Stage (MIS) 3 between 57.3 and 27.82 thousand years ago (kya; Table S5), all associated with Neanderthals and all predating the arrival of Modern Humans in the area. An occasional use of birds for food cannot be ruled out as evidence of burning, human tooth-marks and cut-marks on coracoids, humeri, tibiotarsi and tarsometatarsi have been observed. These could be a response to a subsequent secondary action and are minor in comparison to feather extraction.

**Figure 2:**
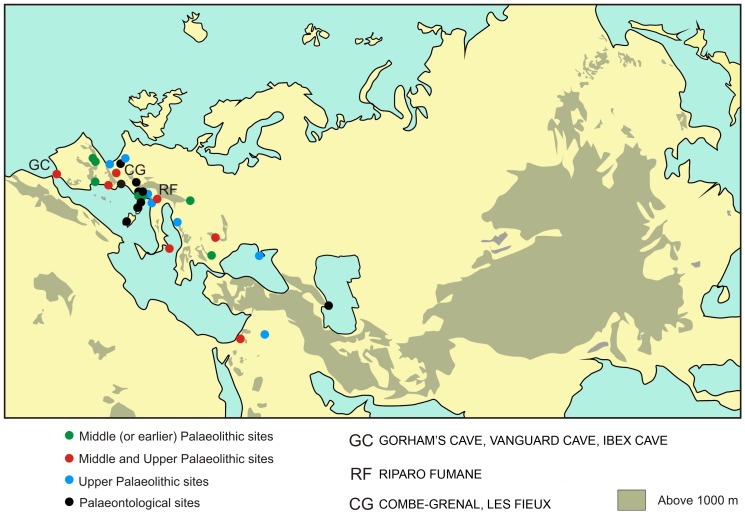
Distribution of archaeological and paleontological sites with 50% or more of the suite of 18 raptor-corvid species identified in the text. Green: Middle (or earlier) Palaeolithic sites; Red: Middle and Upper Palaeolithic Sites; Blue: Upper Palaeolithic Sites; Black: paleontological sites. GC: Gorham's Cave, Vanguard Cave and Ibex Cave; RF: Riparo Fumane; CG: Combe-Grenal and Les Fieux.

To answer our fourth question, whether the observations from the Gibraltar caves represented a local or geographically wider phenomenon, we returned to the evidence from Riparo Fumane [11], almost 2000 kilometres from Gibraltar. In Figure 2 we have plotted the location of Middle and Upper Palaeolithic and palaeontological sites with at least half of the suite of 18 species identified in Table S1; we also added Riparo Fumani (with fewer than half of the species). The results show a clear concentration across the western mid-latitude belt, a topographically heterogeneous region well suited for many scavenging raptors and corvids [7], [17]. The similarity between Middle and Upper Palaeolithic sites may indicate behavioural convergence by two hominins within the same region but separated temporally, or they may instead suggest a case of the transmission of a behavioural association from one group to another or even of shared behavioural ancestry. If it was behavioural transmission, then given the temporal precedence of the Neanderthals, it would indicate that the direction of such transmission would have been from Neanderthals to Modern Humans. In any case, the evidence from Gorham's Cave at least, shows that Neanderthals were capable of this behaviour in the absence of Modern Humans.

## Discussion and Conclusions

The strong relationship between Neanderthals, corvids and raptors requires explanation as does the clear evidence of direct action on the bird bones. If this processing of raptors and corvids by Neanderthals had been related to consumption, then we would have expected a concentration of anthropic marks in parts of the anatomy linked to the fleshy regions of the body (e.g. the sternum which holds the large pectoral muscles). Instead, it is the wing bones, low in meat but anchors for the large flight feathers, which were processed. The overrepresentation of raptor and corvid wing bones in Neanderthal sites cannot thus be interpreted in any way other than the use of their feathers. This is supported by the statistically significantly high proportion of individual wing bones (Goodness of Fit, G_3_ = 139.849, p<0.0001; Table S6) and the fact that these had a statistically significant higher frequency of anthropic marks than other bones (Goodness of Fit, G_2_ = 29.2568, p<0.0001; Table S6). Within the wing bones, humeri and ulnae – bones that support the large flight feathers - appeared to have the highest frequency of anthropic marks (Table S2 and Text S3). The carpo-metacarpi - also supporting flight feathers - might not, we suspect, require as much processing because of their small size, and this may explain the relatively low proportion with anthropic marks. Support that the processing by Neanderthals involved feather removal, and not food, comes from the observation that raptors and corvids are not regularly eaten in any culture, confirmed by the lack of data of corvid or raptor consumption in the ethnographic literature. Feathers as such are not edible either, and they are rapidly disintegrated by feather-degrading bacteria in the soil [24]; thus their use for bedding on cave floors is precluded. The most parsimonious explanation for feather use by Neanderthals would be the same as for tribal Modern Humans: ornaments on their heads and bodies.

Why were dark raptor and corvid feathers selected preferentially over others? These bird species are related to rocky outcrops for nesting and roosting and savannah-like habitats for foraging [7], [17]. They would have therefore been familiar to the Neanderthals and a part of their daily lives; opportunities for obtaining feathers from live birds, at nests or roosts, or from individuals that died and fell to the ground would have been plentiful. They may even have shared the same food resources, as both humans and these scavengers would have coincided around ungulate carcasses. These birds may well have acted as indicators of freshly dead animals to the Neanderthals. Carcasses would have become focal points of convergence for large numbers of vultures, other raptors and corvids, as they still do today. These would have been ideal conditions allowing the Neanderthals the possibility, which would have necessitated a degree of planning and anticipation, of capturing the large birds as they gorged themselves. The behaviour might therefore have originated in the practice of following large birds to fresh carcasses for food. The apparent selection for feathers of specific color, that our results show, adds yet another dimension, requiring sophisticated cognitive processes, to the demonstrated non-random use of feathers.

Lacking previous examples of feather use by Neanderthals, except the valuable recent suggestions by Peresani *et al.* [11], we have reviewed use of feathers by the only surviving *Homo* species Modern Humans, *H. sapiens*. Current or historic use of feathers by Modern Humans is widespread and spans practically every culture that has been studied, including modern western civilization as well as numerous tribal peoples in every permanently inhabited continent (Table S7). This pattern of feather use for adornment appears to be part of the universal human psyche. The Neanderthals clearly shared this invariant behaviour [25] with Modern Humans, suggesting that it may have been a common characteristic of the two lineages, although we cannot determine if one learnt the behaviour from the other or if it was, instead, present in the common ancestor.

Focusing on tribal examples, and assuming they may represent ancestral traditions, we observe that in a majority of cases the use of feathers is ornamental, in the form of headdresses [26], cloth decorations, as in skirts or belts, or even full feather cloaks or capes [27], as those worn by Hawaiian or Maori chiefs. A common characteristic of ornamentation, of which jewellery is the best example, tends to require valuable items that are not easy to replace. Feathers as ornaments seem to follow this rule, common to any biological signal, that is, they are costly to produce or to maintain [28]. The bird species used by humans, such as the Golden Eagle *Aquila chrysaetos* in the case of the Amerindians, were either scarce in the environment [29] or many individuals were needed to produce the elaborate feathered ornaments, as was the case for the red and yellow birds used in Hawaiian capes, in which thousands of individuals were killed to make a single garment [30].

The use of feathers, or the application of other species trophies as adornment on the body, is an exclusively human trait. Feather adornments, however, are not the earliest cases of ornamentation in humans. For Modern Humans, ochre pigment use has been suggested as the first manifestation of symbolic behaviour, in South Africa over 160 thousand years ago [31]; it has been heralded as evidence of the transition to “modernity” in humans. Recently, similar evidence of pigment use has been found in the case of early Neanderthals at Maastricht-Belvédère, Netherland (200–250 kya) [32]. With more recent chronologies, the use of manganese and iron oxides by Late Pleistocene Neanderthals seem to be documented from at least 60 kya onward [33], [34], [35]. In spite of this, the absence of beads, portable figurines or cave art in Neanderthal sites continues to be cited as evidence of their inferior cognitive capacities [36].

That Neanderthals shared this uniquely human trait of feather ornamentation with Modern Humans, provides a further bridge that brings them closer to each other. Recent evidence seems to have resolved the question of Neanderthal-Modern Human gene interchange [37], showing that such exchange in all likelihood occurred in the course of the history of the two lineages. The biological differences between the two could therefore not have been as great as previously envisaged if they were able to interbreed. But the debate of cognitive differences remains open. Discussion of the cognitive abilities of the Neanderthals has a protracted history which came to the fore with the debate on whether ornamentation found associated with Neanderthals in France was autochthonous or was instead the product of acculturation from Modern Humans or trade with them [38], [39]. This debate continues to generate controversy [40], [41] and leaves the question of Neanderthal cognitive capacities unresolved.

The results presented here show that extraction of feathers from birds by Neanderthals was a temporally and geographically widespread phenomenon. The results are reinforced by evidence of repetition of this behaviour across a substantial time period of thousands of years in Gibraltar. The earliest observation of this behaviour in Gibraltar preceded the arrival of Modern Humans in Europe by several thousand years. There is therefore no possibility that the practice was acquired from Modern Humans. Thus Neanderthals, though different in a number of ways from Modern Humans had comparable cognitive capacities that included symbolic expression. The observed behavioural differences between them therefore have to be related to distinct cultural trajectories, as would have been the case between different Modern Human populations [42], [43].

We have shown that Neanderthals were associated with raptors and corvids of particular characteristics (dark remiges, scavenging or colonial cliff nesters) across the entire geographical space of the Palearctic and they directly processed their bones for their feathers. In this respect they were distinctly human. The absence of parietal art in caves occupied by Neanderthals, and also of bone and shell ornaments, is a key argument cited in support of the superior cognitive capacities of Modern Humans. Our results put this long-standing contention in doubt, by providing strong evidence that Neanderthals simply used media, other than cave walls, to express themselves.

## Supporting Information

Table S1 **Database of Pleistocene Palearctic sites based on fossil bird sites catalogued by Tyrberg [Nuttal Ornithol. Club, Cambridge, Mass., 27, 1998/** http:/web.telia.com/-u11502098/pleistocene.html **, 2008].** This database includes all raptor and corvid species as well as corresponding archaeological and paleontological attribution.
(XLS)

Table S2 **NISP by skeletal element and taxa from Gibraltar sites.**
(DOC)

Table S3 **Number of bird bones from the Gibraltar sites with cut-marks.**
(DOC)

Table S4 **Correlation (** ***r Pearson*** **) between maximum bone density of several skeletal elements and main represented species (** ***Pyrrhocorax pyrrhocorax)*** **at Gorham's Cave according to Minimal Number of Elements (MNE).**
(DOC)

Table S5 **Radiocarbon and ESR dates for the Gibraltar cave sites which show evidence of bird processing by Neanderthals.**
(DOC)

Table S6 **Taxonomical representation in Palaeolithic sites (Middle and Upper) compared to paleontological sites, tested by chi-square.** Note species names follow sequence of first three letters of genus and species names -Refer to Table 1 and Table S1-. Fox example, PERAPI is *Pernis Apivorus*.
(XLS)

Table S7 **Current or historic use of feathers by Modern Humans (including modern western civilization and tribal peoples).** Data from Ferraro-Dorta S, Xavier Cury M (2000) *A plumária indígena brasileira no Museu de Arqueologia e Etnologia da USP*. Brazil: Imprensa Oficial SP. 535 p. and Biebuyck DP, Van den Abbeele N (1984) *The power of Headdresses: a cross-cultural study of forms and functions*: Brussels, Belgium: Tendi S.A. 293 p.
(XLS)

Text S1 **Analysis of colour of remiges among raptors and corvids in Palaeolithic and paleontological sites across the Palearctic.**
(DOC)

Text S2 **Taphonomical methods.**
(DOC)

Text S3 **Statistical Analysis of the skeletal representation from the Gibraltar bird remains and of cut-marked bones.** 1) Wing versus leg and axial skeleton bones; 2) individual wing and leg bones and; 3) cut-marks on wing and leg bones.
(DOC)
