# Correction: Birds of a Feather: Neanderthal Exploitation of Raptors and Corvids

**DOI:** 10.1371/annotation/5160ffc6-ec2d-49e6-a05b-25b41391c3d1

**Published:** 2012-10-12

**Authors:** Clive Finlayson, Kimberly Brown, Ruth Blasco, Jordi Rosell, Juan José Negro, Gary R. Bortolotti, Geraldine Finlayson, Antonio Sánchez Marco, Francisco Giles Pacheco, Joaquín Rodríguez Vidal, José S. Carrión, Darren A. Fa, José M. Rodríguez Llanes

There is an error in the funding statement.

The correct statement is: The excavations and scientific research associated with Gorham's Cave have been funded by the Government of Gibraltar and, additionally between 2002–04, by the European Community - Program Interreg IIIB: 2002-02-4, 1-U-048 -. R. Blasco and J. Rosell are part of the following projects: 2009 SGR 188 of Generalitat de Catalunya and CGL2009-12703-C03-02 of the Spanish Goverment. A. Sánchez Marco belongs to the CGL2011-28681 project of the Spanish Government, and J.S. Carrión to CGL-2009-06988/BOS-FEDER. The funders had no role in study design, data collection and analysis, decision to publish, or preparation of the manuscript. 

