# Supplementary material for: Birds of a Feather: Neanderthal Exploitation of Raptors and Corvids
Source: PLoS One. 2012 Sep 17;7(9):e45927. doi: 10.1371/journal.pone.0045927 (PMC3444460; doi:10.1371/journal.pone.0045927)
Supplement: Table S2 — NISP by skeletal element and taxa from Gibraltar sites. (DOC) [file pone.0045927.s002.doc]

**Table S2. NISP by skeletal element and taxa from Gibraltar sites.**

|  |  | Coracoid | Humerus | Ulna | Radius | Cmc | Femur | Tbt | Tmt | Ax | Total |
| --- | --- | --- | --- | --- | --- | --- | --- | --- | --- | --- | --- |
| Gorham’s Cave | *Accipiter gentilis* |  |  |  |  |  |  |  |  | 2 | 2 |
|  | *Accipiter nisus* | 2 | 1 | 3 |  |  | 1 | 1 | 1 | 2 | 11 |
|  | *Aquila chrysaetos* |  |  | 2(1) |  | 1 |  |  |  | 2 | 5(1) |
|  | *Aquila* sp. |  |  |  |  |  |  |  |  | 3 | 3 |
|  | *Buteo buteo* | 1 |  |  |  |  | 2 |  |  |  | 3 |
|  | *Corvus corax* |  |  |  |  |  | 1 |  |  |  | 1 |
|  | *Corvus corone* | 2 | 1 | 4 |  |  |  |  |  | 2 | 9 |
|  | *Corvus corone/frugilegus* | 2 | 1 |  |  | 1 | 1 |  |  | 2 | 7 |
|  | *Corvus monedula* | 1 | 8 | 14 |  | 6 | 6 | 12 | 2 | 9 | 58 |
|  | *Falco naumanni* | 5 | 3 | 3 |  | 2 |  | 7 | 8 |  | 28 |
|  | *Falco peregrinus* |  |  |  |  | 2 |  |  | 1 | 1 | 4 |
|  | *Falco subbuteo* |  | 1 |  |  |  |  |  |  |  | 1 |
|  | *Falco tinnunculus* | 5 | 4 | 6 |  | 5 | 2 | 3 | 8 | 1 | 34 |
|  | *Gyps melitensis*/*fulvus* |  | 1 | 1 |  | 2 | 1(1) |  |  | 9 | 14(1) |
|  | *Gyps fulvus* |  |  |  |  |  |  |  |  | 4 | 4 |
|  | *Gyps/aegypius* |  | 1 |  |  |  |  |  |  |  | 1 |
|  | *Milvus migrans* |  |  |  |  |  |  | 1(1) |  |  | 1(1) |
|  | *Milvus milvus* | 4(1) | 3(1) | 4(1) | 1(1) | 1(1) | 1 | 6 | 2 |  | 22(5) |
|  | *Milvus* sp. | 1 |  | 1 | 1 |  |  | 2 | 1 | 2 | 8 |
|  | *Pica pica* | 2 |  | 2 |  |  |  | 1 | 2 | 2 | 9 |
|  | *Pyrrhocorax graculus* | 9 | 20(6) | 13(6) | 1 | 4 | 7 | 6 | 6 | 7 | 73(12) |
|  | *Pyrrhocorax pyrrhocorax* | 27(2) | 31(6) | 36(9) |  | 24 | 19 | 18 | 18 | 7 | 180(17) |
|  | *Pyrrhocorax* sp. |  | 4(1) | 2 |  | 1 |  |  |  |  | 7(1) |
|  | Unident. Bird of prey |  |  |  |  |  |  |  |  | 1 | 1 |
|  | Total | 61(3) | 79(14) | 91(17) | 3(1) | 49(1) | 41(1) | 57(1) | 49 | 56 | 486(38) |
| Vanguard Cave | *Accipiter gentilis* | 1 |  |  |  | 1 |  |  |  |  | 2 |
|  | *Accipiter nisus* |  | 2(1) | 1 |  |  | 1 |  | 1 | 1 | 6(1) |
|  | *Accipiter* sp. |  |  |  |  |  |  |  |  | 2 | 2 |
|  | *Aquila* sp./*haliaeetus* sp. |  |  |  |  |  |  |  |  | 1 | 1 |
|  | *Buteo* sp. |  |  |  |  |  |  |  |  | 1 | 1 |
|  | *Circus cyaneus* |  |  |  |  | 1 |  |  |  |  | 1 |
|  | *Corvus corax* |  |  | 1 |  |  |  |  |  | 1 | 2 |
|  | *Corvus monedula* | 2 | 2 | 3 |  |  | 1 | 4 | 3 | 1 | 16 |
|  | *Falco naumanni* |  |  | 2 |  |  |  | 2 | 2 |  | 6 |
|  | *Falco tinnunculus* |  | 2 | 1 |  | 1 |  |  |  |  | 4 |
|  | *Falco* sp. |  |  |  |  |  |  |  |  | 2 | 2 |
|  | *Falco/accipiter* sp. |  |  |  |  |  |  |  |  | 1 | 1 |
|  | *Gyps fulvus* | 1 | 3 | 3(2) |  |  | 1 |  |  | 8 | 16(2) |
|  | *Gyps* sp. |  | 1 |  |  |  |  |  |  |  | 1 |
|  | *Gyps/aegypius* |  | 1 |  |  |  | 1 |  |  | 1 | 3 |
|  | *Haliaeetus albicilla* |  |  |  |  | 1 |  |  |  |  | 1 |
|  | *Hieraaetus fasciatus* |  |  |  |  |  |  |  | 1 |  | 1 |
|  | *Milvus* sp. | 1 |  |  |  |  |  |  |  |  | 1 |
|  | *Pyrrhocorax graculus* | 2 |  |  |  |  |  | 1 |  |  | 3 |
|  | *Pyrrhocorax pyrrhocorax* |  | 2(1) | 5 |  | 5 |  | 3 | 1 | 1 | 17(1) |
|  | *Pyrrhocorax* sp. |  | 1 |  |  |  |  |  |  |  | 1 |
|  | Unident. Bird of prey |  |  |  |  |  |  |  |  | 3 | 3 |
|  | Total | 7 | 14(2) | 16(2) | - | 9 | 4 | 10 | 8 | 23 | 91(4) |
| Ibex Cave | *Aquila* sp. |  |  |  |  |  |  |  |  | 1 | 1 |
|  | *Corvus corax* |  |  | 1 |  | 1 |  |  | 1 |  | 3 |
|  | *Corvus corone* | 1 |  |  |  |  | 1 |  |  |  | 2 |
|  | *Gypaetus barbatus* |  |  |  |  |  |  |  | 1 |  | 1 |
|  | *Pyrrhocorax pyrrhocorax* | 3 |  |  |  | 2 | 2(1) | 1 | 9(3) | 3 | 20(4) |
|  | Total | 4 | - | 1 | - | 3 | 3(1) | 1 | 11(3) | 4 | 27(4) |

Number between brackets shows NISP with anthropogenic damage. Cmc: carpometacarpus; Tbt: Tibiotarsus; Tmt: Tarsometatarsus; Ax: axial elements (vertebrae and ribs).
