# Supplementary material for: Birds of a Feather: Neanderthal Exploitation of Raptors and Corvids
Source: PLoS One. 2012 Sep 17;7(9):e45927. doi: 10.1371/journal.pone.0045927 (PMC3444460; doi:10.1371/journal.pone.0045927)
Supplement: Table S3 — Number of bird bones from the Gibraltar sites with cut-marks. (DOC) [file pone.0045927.s003.doc]

**Table S3. Number of bird bones from the Gibraltar sites with cutmarks.**

| **Site** | **Species** | **Site reference** | **Skeletal**  **element** | **No.striae**  **by group** | **Distribution** | **Cutmark**  **type** | **Portion location** | **Side location** | **Orient.** | **Delin.** |
| --- | --- | --- | --- | --- | --- | --- | --- | --- | --- | --- |
| Gorham’s Cave | *Aquila chrysaetos* | GOR’03/A9/NIV/70 | Ulna | 2 | cl | inc | middle diaphysis | cranial | tr | str |
|  | *Gyps melitensis/fulvus* | GOR’05/AA4/NIV/16 | Femur | 2 | cl | inc | prox. diaphysis | lateral | tr | str |
|  | *Milvus migrans.* | GOR’00/B9/NIV/GEN(280) | Tbt | 2 | cr | inc | middle diaphysis | caudal | obl | curv- str |
|  | *Milvus milvus* | GOR’00/B8/NIV/205 | Radius | 2 | cr | inc | distal diaphysis | cranial | obl-tr | str |
|  |  | GOR’05/A6/NIV/127 | Ulna | 2-3 | cl | inc | distal end | caudal | obl | curv |
|  |  | GOR’03/A10/NIV/GEN(300) | Coracoid | 2 | cl | inc | middle diaphysis | dorsal | obl | curv |
|  |  | GOR'00/B10/NIV/GEN AV0004 | Cmc | 2 | cl | inc | prox. end | dorsal | obl | str |
|  | *Pyrrhocorax graculus* | GOR’00/B5/NIV/57 | Ulna | 3 | cl | inc | prox. diaphysis | lateral | obl | str |
|  |  | GOR’03/A10/NIV/GEN(288) | Ulna | 4 | cl | inc | middle diaphysis | lateral | tr | str |
|  |  | GOR’05/AA4/NIV/38 | Humerus | 2 | cl | inc | prox. diaphysis | caudal | long | str |
|  |  | GOR’03/A9/NIV/72 | Humerus | 4 | cl | inc | distal diaphysis | caudal | tr | str |
|  |  | GOR’03/A11/NIV/91 | Ulna | 8 | cl | scr | middle to prox. diaphysis | medial | obl | str |
|  |  | GOR’05/AA4/NIV/170 | Humerus | 2 | cl | inc | prox. diaphysis | caudal | tr | str |
|  |  | GOR’03/A9/NIV/80 | Humerus | 2 | cl | inc | prox. end | cranial | tr | curv |
|  |  | GOR’05/AA4/NIV/52 | Humerus | 2-1 | cl-is | inc | prox. end | cranial | long | str |
|  |  | GOR’03/A10/NIV/GEN(288) | Humerus | 1-2-1 | is-cl | inc | middle diaphysis | lateral | tr | str |
|  | *Pyrrhocorax pyrrhocorax* | GOR’96 NO.299 | Humerus | 3 | is | inc | prox. diaphysis | medial | obl | curv |
|  |  | GOR’96 NO.103 | Ulna | 1 | is | inc | prox. diaphysis | lateral | obl | str |
|  |  | GOR’96 NO.87 | Humerus | 3 | cl | inc | distal diaphysis | caudal | long | curv |
|  |  | GOR’99/aa2/NIV/GEN(230) | Humerus | 5 | cl | scr | middle diaphysis | caudal | long | str |
|  |  | GOR’99/aa2/NIV/11 | Humerus | 2-3 | cl | inc | middle diaphysis | caudal | tr | str |
|  |  | GOR’99/aa2/NIV/44 | Ulna | 2 | cr | inc | prox. diaphysis | medial | obl-tr | str |
|  |  | GOR’99/aa2/NIV/44 | Ulna | 2 | cl | inc | middle diaphysis | cranial | obl | str |
|  |  | GOR’96 No.39 | Coracoid | 1 | is | inc | prox. diaphysis | cranial | obl | str |
|  |  | GOR’03/A10/NIV/GEN (300) | Humerus | 2 | cl | inc | prox diaphysis | cranial | tr | curv |
|  |  | GOR’99/aa2/NIV/GEN(230) | Ulna | 3 | cl | scr | distal end | medial | obl | curv |
| Ibex Cave | *Pyrrhocorax pyrrhocorax* | Ibex 94 No.166 | Femur | 3 | is | inc | middle diaphysis | lateral | tr | str |
|  |  | Ibex 94 No.24 | Tmt | 2 | is | inc | middle diaphysis | posterior | obl | str |
|  |  | Ibex 94 No.152 | Tmt | 6 | cr | scr | middle diaphysis | lateral | tr | curv |
|  |  | Ibex 94 No.61 | Tmt | 3 | cl | scr | middle diaphysis | dorsal | tr | str |
| Vanguard Cave | *Gyps fulvus* | Van 96 No.209A | Ulna | 1-4 | is - cl | inc | distal diaphysis | cranial | obl | str |
|  |  | Van 96 No.117 | Ulna | 2-2 | is | inc | distal diaphysis | caudal-cranial | obl | str |
|  | *Pyrrhocorax pyrrhocorax* | Van 95 No. 114 | Humerus | 1 | is | inc | prox. diaphysis | caudal | obl | str |

Cmc: carpometacarpus; Tbt: Tibiotarsus; Tmt: Tarsometatarsus; cl= clustered; cr= crossed; is= isolated; inc= incisions; scr= scrapes; obl: oblique; long: longitudinal; tr: transverse; str= straight, curv= curved.
