# Supplementary material for: Birds of a Feather: Neanderthal Exploitation of Raptors and Corvids
Source: PLoS One. 2012 Sep 17;7(9):e45927. doi: 10.1371/journal.pone.0045927 (PMC3444460; doi:10.1371/journal.pone.0045927)
Supplement: Table S4 — Correlation ( r Pearson ) between maximum bone density of several skeletal elements and main represented species ( Pyrrhocorax pyrrhocorax) at Gorham's Cave according to Minimal Number of Elements (MNE). (DOC) [file pone.0045927.s004.doc]

**Table S4. Correlation (*r Pearson*) between maximum bone density of several skeletal elements and main represented species (*Pyrrhocorax pyrrhocorax)* at Gorham’s Cave according to Minimal Number of Elements (MNE).**

|  | ***Phalacrocorax auritus*** | | ***Branta canadensis*** | | ***Anas platyrrhynchus*** | |
| --- | --- | --- | --- | --- | --- | --- |
|  | **Bone density** | **MNE** | **Bone density** | **MNE** | **Bone density** | **MNE** |
| Cranium | 0.526 | 0 | 0.191 | 0 | 0.314 | 0 |
| Dentary | 0.872 | 0 | 0.324 | 0 | 0.322 | 0 |
| Sternum | 0.283 | 0 | 0.24 | 0 | 0.26 | 0 |
| Furculum | 0.243 | 0 | 0.516 | 0 | 0.404 | 0 |
| Scapula | 0.409 | 0 | 0.617 | 0 | 0.583 | 0 |
| Coracoid | - | 27 | 0.458 | 27 | 0.569 | 27 |
| Humerus | 0.588 | 31 | 0.403 | 31 | 0.484 | 31 |
| Ulna | 0.706 | 34 | 0.511 | 34 | 0.599 | 34 |
| Radius | 0.595 | 0 | 0.728 | 0 | 0.464 | 0 |
| Carpometacarpus | 0.628 | 24 | 0.548 | 24 | 0.758 | 24 |
| Synsacrum | 0.410 | 0 | 0.174 | 0 | 0.219 | 0 |
| Femur | 0.461 | 19 | 0.364 | 19 | 0.381 | 19 |
| Tibotarsus | 0.453 | 18 | 0.576 | 18 | 0.503 | 18 |
| Tarsometatarsus | 0.532 | 18 | 0.433 | 18 | 0.241 | 18 |
| r Pearson | 0.35244 | | 0.21571 | | 0.54486 | |

Bone density data from value estimated by Broughton et al. [1] for some bird taxa.

References

**1. Broughton JM**, Mullins D, Ekker T (2007) Avian resource depression or intertaxonomic variation in bone density? A test with San Francisco Bay avifaunas*.* J Archaeol Sci 34: 374-391.
