# Supplementary material for: Birds of a Feather: Neanderthal Exploitation of Raptors and Corvids
Source: PLoS One. 2012 Sep 17;7(9):e45927. doi: 10.1371/journal.pone.0045927 (PMC3444460; doi:10.1371/journal.pone.0045927)
Supplement: Table S5 — Radiocarbon and ESR dates for the Gibraltar cave sites which show evidence of bird processing by Neanderthals. (DOC) [file pone.0045927.s005.doc]

**Table S5. Radiocarbon and ESR dates for the Gibraltar cave sites which show evidence of bird processing by Neandertals.**

GORHAM’S CAVE MOUSTERIAN RADIOCARBON DATES FROM THE UPPER GALLERY (Finlayson et al., 2006)

| Lab. Code | Material | Conventional  Radiocarbon age  yr BP | δ^13^C (‰) | Cal. Age yr BP  IntCal09 (2σ) |
| --- | --- | --- | --- | --- |
| B-196782 | charcoal | 23,360±160 | -22.4 | 27,820–28,560 |
| B-185345 | charcoal | 23,780±270 | -25.0 | 27,990–29,300 |
| B-196775 | charcoal | 24,010±160 | -24.0 | 28,400–29,330 |
| B-196785 | charcoal | 26,070±180 | -25.6 | 30,440–31,120 |
| B-196773 | charcoal | 26,400±220 | -23.2 | 30,620–31,290 |
| B-185344 | charcoal | 27,020±240 | -25.0 | 31,050–31,620 |
| B-185346 | charcoal | 27,280±220 | - | 31,140–31,890 |
| B-196770 | charcoal | 28,170±240 | -25.9 | 31,630–33,130 |
| B-196784 | charcoal | 28,360±240 | -26.1 | 31,810–33,340 |
| B-196791 | charcoal | 28,570±240 | -25.2 | 31,930–33,930 |
| B-184048 | charcoal | 29,210±190 | -25.2 | 33,300–34,540 |
| B-184049 | charcoal | 29,240±190 | - | 33,320–34,550 |
| B-196779 | charcoal | 29,400±270 | -25.4 | 33,330–34,660 |
| B-196778 | charcoal | 29,720±280 | -24.8 | 33,520–34,890 |
| B-196786 | charcoal | 29,910±300 | -24.7 | 33,690–35,080 |
| B-196792 | charcoal | 30,310±310 | -24.7 | 34,460–36,180 |
| B-196776 | charcoal | 30,560±360 | -24.5 | 34,570–36,270 |
| B-184045 | charcoal | 31,110±230 | -23.7 | 35,030–36,340 |
| B-196768 | charcoal | 31,290±340 | -25.8 | 35,050–36,510 |
| B-196787 | charcoal | 31,480±370 | -23.7 | 35,090–36,620 |
| B-196772 | charcoal | 31,780±360 | -23.1 | 35,180–36,900 |
| B-196769 | charcoal | 31,850±380 | -23.5 | 35,160–37,080 |
| B-196789 | charcoal | 32,100±400 | -24.5 | 35,300–37,710 |
| B-196771 | charcoal | 32,560±390 | -25.1 | 36,460–38,460 |

GORHAM’S CAVE MOUSTERIAN RADIOCARBON DATES FROM THE ENTRANCE (Pettitt and Bailey, 2000)

| Lab. Code | Material | Conventional  Radiocarbon age  yr BP | δ^13^C (‰) | Cal. Age yr BP  IntCal09 (2σ) |
| --- | --- | --- | --- | --- |
| OxA-7979 | charcoal | 23,800±300 | -21.7 | 27,980–29,350 |
| OxA-6997 | burnt bone | 25,680±140 | -21.2 | 30,240–30,880 |
| OxA-7792 | charcoal | 28,680±120 | -24.5 | 32,580–33,570 |
| OxA-7077 | charcoal | 29,250±325 | -24.7 | 33,110–34,650 |
| OxA-7110 | charcoal | 29,250±375 | -24.4 | 32,970–34,690 |
| OxA-7075 | charcoal | 29,800±350 | -27.3 | 33,480–35,030 |
| OxA-7074 | charcoal | 30,200±350 | -24.2 | 33,950–36,170 |
| OxA-7076 | charcoal | 30,250±350 | -25.2 | 34,050–36,180 |
| OxA-8541 | charcoal | 31,900±700 | -24.9 | 34,970–38,380 |
| OxA-7857 | charcoal | 32,280±210 | -22.8 | 36,380–37,380 |
| OxA-7791 | charcoal | 42,200±550 | -23.9 | 44,640–46,360 |
| OxA-8542 | charcoal | 42,800±1050 | -24.4 | 44,400–48,500 |
| OxA-8525 | charcoal | 43,800±650 | -20.9 | 45,580–48,710 |
| OxA-6075 | charcoal | 45,300±850 | -25.2 | 46,680–50,000* |
| OxA-8526 | charcoal | 46,700±950 | -23.7 | date out of range |
| OxA-205 | charcoal | 47,900±1050 | -23.4 | date out of range |
| OxA-7790 | charcoal | 51,700±1650 | -24.5 | date out of range |

VANGUARD CAVE MOUSTERIAN RADIOCARBON DATES (Pettitt and Bailey, 2000)

| Lab. Code | Material | Conventional  Radiocarbon age  yr BP | δ^13^C (‰) | Cal. Age yr BP  IntCal09 (2σ) |
| --- | --- | --- | --- | --- |
| OxA-6998 | charcoal | 41,800±700 | -25.1 | 44,240–46,260 |
| OxA-7389 | charcoal | 45,200±1200 | -25.5 | 46,250–50,000* |
| OxA-6892 | charcoal | 46,900±750 | -22.6 | date out of range |
| OxA-7127 | charcoal | >49,400 | -24.4 | date out of range |
| OxA-6891 | charcoal | 54,000±1650 | -22.1 | date out of range |

Ranges marked with a (*) are suspect due to impingment on the end of the calibration data set.

IBEX CAVE MOUSTERIAN ESR DATES (Rhodes et al., 2000)

| Sample-Lab. Code | Material | Early Uptake  Age (ka) | Linear Uptake  Age (ka) |
| --- | --- | --- | --- |
| Ibex Cave 9415-555A | tooth | 43.3±2.6 | 53.9±3.4 |
| Ibex Cave 9415-555B | tooth | 37.2±2.4 | 45.7±3.1 |
| IC94C10 Unit319-555A1 | tooth | 40.8±2.4 | 54.9±3.2 |
| IC94C10 Unit319-555A2 | tooth | 36.6±2.1 | 50.2±2.8 |
| IC94C10 Unit319-555A3 | tooth | 37.8±2.1 | 50.6±2.9 |
| IC94C10 Unit319-555B | tooth | 37.4±2.1 | 50.9±2.9 |
| IC94C10 Unit319-555C | tooth | 29.3±1.6 | 45.2±2.4 |
| IC94C10 Unit319-555D | tooth | 35.6±2.0 | 46.4±2.6 |
| IC94C10 Unit319-555E | tooth | 34.8±2.0 | 45.7±2.6 |
| IC94C10 Unit319-555F | tooth | 37.2±2.2 | 50.0±3.1 |
| IC94C10 Unit319-555G | tooth | 39.6±2.3 | 52.3±3.2 |
| IC94C10 Unit319-555H | tooth | 37.1±2.3 | 49.2±3.0 |
| IC94C10 Unit319-555I | tooth | 36.1±2.1 | 47.7±2.8 |
| Mean age (ka) |  | 37.1±3.3 | 49.4±3.2 |
