# Supplementary material for: Birds of a Feather: Neanderthal Exploitation of Raptors and Corvids
Source: PLoS One. 2012 Sep 17;7(9):e45927. doi: 10.1371/journal.pone.0045927 (PMC3444460; doi:10.1371/journal.pone.0045927)
Supplement: Text S1 — Analysis of colour of remiges among raptors and corvids in Palaeolithic and paleontological sites across the Palearctic. (DOC) [file pone.0045927.s008.doc]

**Text S1. Analysis of Colour of Remiges among raptors and corvids in Paleolithic and paleontological sites across the Palearctic**

Remige colour was classified according to the following categorisation: d= dark - some feathers are uniformly black or brown or have brown bars (but no white, like the adult Golden Eagle *Aquila chrysaetos*); i = intermediate - this includes birds with light brown or more often spotting or barred patterns so have some white and some dark per feather -some species like the Osprey *Pandion haliaetus* and buteos have white and brown bars or like kestrels have light and dark spots, and a few species have light brown or light bars but not really white and not really dark; and m=mix where some feathers are white (e.g. primaries) and some are black (e.g. secondaries) - some spp like the Magpie *Pica pica* have a mix of light and dark feathers on the wing. Given the small sample of mix category the analysis was limited to dark versus intermediate types. The first analysis (a) compares Paleolithic versus paleontological sites; the second (b) analysis compares Upper versus Middle Paleolithic sites.

(a)

| **Remige dark vs inter minus mix * Overreppalaeo Crosstabulation** | | | | | |
| --- | --- | --- | --- | --- | --- |
|  | | | Overreppalaeo | | Total |
|  |  |  | no | yes |  |
| Remige dark vs inter minus mix | dark | Count | 4 | 12 | 16 |
|  |  | % within remige dark vs inter minus mix | 25.0% | 75.0% | 100.0% |
|  | inter | Count | 18 | 7 | 25 |
|  |  | % within remige dark vs inter minus mix | 72.0% | 28.0% | 100.0% |
| Total | | Count | 22 | 19 | 41 |
|  |  | % within remige dark vs inter minus mix | 53.7% | 46.3% | 100.0% |

| **Chi-Square Tests** | | | | | |
| --- | --- | --- | --- | --- | --- |
|  | Value | df | Asymp. Sig. (2-sided) | Exact Sig. (2-sided) | Exact Sig. (1-sided) |
| Pearson Chi-Square | 8.667^a^ | 1 | .003 |  |  |
| Continuity Correction^b^ | 6.880 | 1 | .009 |  |  |
| Likelihood Ratio | 8.976 | 1 | .003 |  |  |
| Fisher's Exact Test |  |  |  | .005 | .004 |
| N of Valid Cases | 41 |  |  |  |  |
| a. 0 cells (.0%) have expected count less than 5. The minimum expected count is 7.41. | | | | | |
| b. Computed only for a 2x2 table | | | | | |

(b)

| **Remige dark vs inter minus mix * Overrepmid Crosstabulation** | | | | | |
| --- | --- | --- | --- | --- | --- |
|  | | | Overrepmid | | Total |
|  |  |  | no | yes |  |
| Remige dark vs inter minus mix | dark | Count | 9 | 7 | 16 |
|  |  | % within remige dark vs inter minus mix | 56.3% | 43.8% | 100.0% |
|  | inter | Count | 23 | 2 | 25 |
|  |  | % within remige dark vs inter minus mix | 92.0% | 8.0% | 100.0% |
| Total | | Count | 32 | 9 | 41 |
|  |  | % within remige dark vs inter minus mix | 78.0% | 22.0% | 100.0% |

| **Chi-Square Tests** | | | | | |
| --- | --- | --- | --- | --- | --- |
|  | Value | df | Asymp. Sig. (2-sided) | Exact Sig. (2-sided) | Exact Sig. (1-sided) |
| Pearson Chi-Square | 7.278^a^ | 1 | .007 |  |  |
| Continuity Correction^b^ | 5.341 | 1 | .021 |  |  |
| Likelihood Ratio | 7.287 | 1 | .007 |  |  |
| Fisher's Exact Test |  |  |  | .017 | .011 |
| N of Valid Cases | 41 |  |  |  |  |
| a. 1 cells (25.0%) have expected count less than 5. The minimum expected count is 3.51. | | | | | |
| b. Computed only for a 2x2 table | | | | | |
