# Supplementary material for: Birds of a Feather: Neanderthal Exploitation of Raptors and Corvids
Source: PLoS One. 2012 Sep 17;7(9):e45927. doi: 10.1371/journal.pone.0045927 (PMC3444460; doi:10.1371/journal.pone.0045927)
Supplement: Text S2 — Taphonomical methods. (DOC) [file pone.0045927.s009.doc]

**Text S2. Taphonomical methods**

Bird remains from Gibraltar sites were analysed at anatomical and taxonomical level and several taphonomical modifications were recorded. To assess completeness of the sample, NISP (Number of Identified Specimens), MNE (Minimum Number of Elements) and MNI (Minimum Number of Individuals) were calculated. A bivariant test (r Pearson) was used to check the existence of a possible differential preservation of certain anatomical portions according to bone density [1]. For calculation of this, bone density data estimated by Broughton et al. [2] were used.

Surface alterations generated by the hominids were treated at both macroscopic and microscopic level. For microscopic study an Olympus Europe SZ11 (magnification up to 120) and an Environmental Scanning Electron Microscope (FEI Quanta 600) were used. Damage observed on birds remains included cut-marks, fresh bone breakage, overextending (arrachement and peeling) burning and human tooth-marks.

Cut-marks were identified according to criteria established by Binford, [3] Potts and Shipman [4], Shipman [5], Shipman and Rose [6], Shipman et al. [7] and Bromage and Boyde [8]. Two types of cut-marks were identified and grouped into incisions and scrapes. The incisions are striations with a linear outline of variable length, width, and depth. The incisions have a V-shaped section and display internal microstriation [4]. In some cases Hertzian cones [7], shoulder effects and barbs [6] were found. The scrape marks are shallow sub-parallel striae [9] caused when a stone tool is dragged transversally along the length of the bone. The analysis of cut-marks took into account the number of striations, location on the anatomical element, orientation regarding the longitudinal axis of the bone (oblique, longitudinal, transverse) and distribution over the surface (isolated, clustered, crossed).

Bone breakage was analyzed and classified as either being old (at or near the time of deposition) or new (during or after excavation) [10]. This last type was well defined by colour changes in the section of bone. Anyway, the outline (transverse, curved/V-shaped, longitudinal) and the fracture angle (oblique, right, mixed) were also taken into account following to criteria described by Villa and Mahieu [11]. This analysis allowed us to distinguish the bone state when it was fractured. Fresh bone breakage on bird remains can be the result of several processes, such as disarticulation or removal of marrow, fat and cartilage. These phenomena generate certain modifications (peeling and arrachement], which were here grouped under over-extending damage. Peeling is defined as a roughened surface with parallel grooves and fibrous texture [12]. Arrachement is the loss of bone cortical tissue related to the disarticulation and affect mainly to distal ends of humerus and radius and to articular ends of ulna. In the case of humerus, this process often generates one hole on distal ends [13].

Thermal-modifications were also identified [14, 15, 16, 17]. Burning damage documented was here arranged in 6 degrees (from degree 0 or unburned to degree 5 or calcined).

Tooth-marks were identified and compared with the human tooth-marks [13, 18] and with the non-human predator marks [19, 20, 13, 21, 22, 23]. Pits and scores were the main tooth-marks observed [24, 25]. Pits are small marks that superficially penetrate the cortical of the bones. Scores are surface marks with the along-axis more than four times the perpendicular axis. Tooth-mark number and location on bones were recorded. No crenulated edges generated by carnivores were observed [24, 25].

Similarly, digested bones were occasionally documented. These alterations were analyzed following the observations described by several authors [26, 27].

Post-depositional modifications were identified, specifically root-etching and trampling [28, 29]. To assess the influence caused by the agent responsible of these modifications, a classification by degrees was used: Grade 0 or unmodified, Grade 1 or slightly modified, Grade 2 or moderately modified and Grade 3 or highly modified.

References

1. Lyman RL (1994) Vertebrate Taphonomy. Cambridge: University Press. 524 p.

2. **Broughton JM**, Mullins D, Ekker T (2007) Avian resource depression or intertaxonomic variation in bone density? A test with San Francisco Bay avifaunas*.* J Archaeol Sci 34: 374-391.

3. Binford LR (1981) Bones: Ancient Men and Modern Myths. New York: Academic Press. 320 p.

4. Potts R, Shipman P (1981) Cutmarks made by stone tools on bones from Olduvai Gorge, Tanzania. Nature 291: 577-580.

5. Shipman P (1983) Early hominid lifestyle: hunting and gathering or foraging and scavenging? In: Clutton-Brock J, Grigson C, editors. Hunters and Their Prey. Animals and Archaeology, Vol. 1. Oxford: British Archaeological Reports International Series. pp. 31-49.

6. Shipman P, Rose J (1983) Early hominid hunting, butchering and carcass-processing behaviors: approaches to the fossil record. J Anthropol Archaeol 2: 57-98.

7. Shipman P, Fisher DC, Rose J. (1984) Mastodon butchery: microscopic evidence of carcass processing and bone tool use. Paleobiology 10: 358-365.

8. Bromage TG., Boyde A (1984) Microscopic criteria for the determination of directionality cutmarks on bone. Am J Phys Anthrop 65: 359-366.

9. Noe-Nygaard N (1989) Man-made trace fossils on bones. J Hum Evol 4: 461-491.

10. Steadman DW, Plourde A, Burley DV (2002) Prehistoric butchery and consumption of birds in the kingdom of Tonga, South Pacific. J Archaeol Sci 29: 571-584.

11. Villa P, Mahieu E (1991) Breakage patterns of human long bones. J Hum Evol 21: 27-48.

12. White TD (1992) Prehistoric Cannibalism at Mancos 5MTURM-2346. Princeton: University Press. 492 p.

13. Laroulandie V (2000) Taphonomie et archéozoologie des oiseaux en grotte: applications aux sites paléolithiques du Bois Ragot (Vienne), de Combe Saunière (Dordogne) et de La Vache (Ariège). Ph D. Universite Bordeaux-I, France.

14. Shipman P, Foster GF, Schoeninger M (1984). Burnt bones and teeth: and experimental study of colour, morphology, crystal structure and shrinkage. J Archaeol Sci 11: 307-325.

15. Guillon F (1986) Os Brules secs ou frais?. Anthropologie Physique et Archeologie. Paris: CNRS. pp. 191-194.

16. Buikstra J, Swegle M (1989) Bone modification due to burning: experimental evidence. In: Bonnichsen R, Sorg E, editors. Bone Modification. Orono: University of Maine, pp. 247-258.

17. Nicholson RA (1993) A morphological investigation of burnt animal bone and an evaluation of its utility in Archaeology. J Archaeol Sci 20: 411-428.

18. Laroulandie V (2005) Anthropogenic versus non-anthropogenic bird bone assemblages: new criteria for their distinction. In: O’Connor T, editor. Biosphere to Lithosphere: New Studies in Vertebrate Taphonomy. Oxford: Oxbow Books. pp. 25-30.

19. Mayhew DF (1976) Avian predators as accumulators of fossils mammals material. Boreas 6: 25-31.

20. Schmitt DN (1995) The taphonomy of golden eagle prey accumulations at Grean Basin roots. J Ethnobiol 15: 237-256.

21. Bochenski ZM, Tornberg R (2003) Fragmentation and preservation of bird bones in uneaten food remains of the gyrfalcon Falco rusticolus. J Archaeol Sci 30: 1665-1671.

22. Cruz I (2008) Avian and mammalian bone taphonomy in southern continental Patagonia: a comparative approach. Quat. Int. 180: 30-37.

23. Bochenski ZM, Tomek T, Tornberg R, Wertz K (2009) Distinguishing nonhuman predation on birds: pattern of damage done by the white-tailed eagle *Haliaeetus albicilla*, with comments on the punctures made by the golden eagle *Aquila chrysaetos*. J Archaeol Sci 36: 122-129.

24. Haynes G (1980) Evidence of carnivore gnawing on Pleistocene and recent mammalian bones. Paleobiology 6: 341-351.

25. Haynes G (1983) A guide for differentiating mammalian carnivores taxa responsable for gnaw damage to herbivore limb bones. Paleobiology 9: 164-172

26. Bochenski ZM, Tomek T (1997) Preservation of bird bones: erosion versus digestion by owls. Int J Osteoarchaeol 7: 372-387.

27. Bochenski ZM, Huhtala K, Jussila P, Pulliainen E, Tornberg R, Tunkkari PS (1998) Damage to bird bones in pellets of gyrfalcon *Falco rusticolus*. J. Archaeol. Sci. 25: 425-433.

28. Blasco R, Rosell J, Fernández Peris J, Cáceres I, Vergés JM (2008) A new element of trampling: an experimental application on the Level XII faunal record of Bolomor Cave (Valencia, Spain). J Archaeol Sci 35: 1605-1618.

29. Domínguez-Rodrigo M, de Juana S, Galán A, Rodríguez M (2009) A new protocol to differentiate trampling marks from butchery cut marks. J Archaeol Sci 36: 2643-2654.
