# Supplementary material for: Birds of a Feather: Neanderthal Exploitation of Raptors and Corvids
Source: PLoS One. 2012 Sep 17;7(9):e45927. doi: 10.1371/journal.pone.0045927 (PMC3444460; doi:10.1371/journal.pone.0045927)
Supplement: Text S3 — Statistical Analysis of the skeletal representation from the Gibraltar bird remains and of cut-marked bones. 1) Wing versus leg and axial skeleton bones; 2) individual wing and leg bones and; 3) cut-marks on wing and leg bones. (DOC) [file pone.0045927.s010.doc]

**Text S3**

**1. Statistical Analysis of wing versus leg and axial skeleton bones**

Coracoids were left out as they are effectively tied closely to the body that they are effectively not part of the wing. Removal reduced the wing component, thus making the difference with other bones less pronounced. To calculate expected proportion of bones we used the distribution of these bones in a typical bird. There are 4 wing elements 3 leg elements and in the case of axial skeleton, the corrected number is 53: (Cervical vertebra (15), Thoracic vertebra (5), Lumbar vertebra (6), Synsacrum (1), Caudal vertebra (15), Ribs (10), Sternum (1). Therefore the expected proportions for wing-leg and axial are in the ratio of (4x2):(3x2):53 or 11.9% wing, 9.0% leg and 79.1% axial, when in fact the actual % observed was 49.8% wing, 29.1% leg and 15.6% axial.

Therefore the table of observed *vs* expected (necessary for a goodness of fit G test) was:

Wing Leg Axial

Observed 265 184 83

Expected 63.3 47.8 420.8

**G=985.4379 df = 2 P < 0.0001**

**2. Statistical analysis of individual wing and leg bones**

Leg bones are found roughly in proportion to their number:

Femur Tbt Tmt

Observed 48 68 68

Expected 61.3 61.3 61.3

**G= 4.734 df=2 P>.05 so NS but P is not greater than 0.1**

However Wing bones are not in proportion expected:

Humerus Ulna Radius Carp

Observed Ns 93 108 3 62

Expected 66.5 66.5 66.5 66.5

**G=139.849 df=3 P<0.0001**

The low number of radii (significantly the only wing bone with no attachment of long feathers) is indicative although its low frequency may, alternatively, represent poor conservation due to its fragility.

**3. Statistical analysis of cut-marks on wing and leg bones**

Proportions of anthropogenic marks on wing *vs* leg *vs* axial are compared. The prediction is that wing should be greatest.

Wing Leg Axial

N bones total 265 184 83

N anthrop 37 6 0

% anthrop 14.0 3.3 0

43 bones of 532 had marks = 8.1%

if marks were randomly assigned the expected N would be the same % per bone category, or

N Expected 21.5 14.9 6.7

**G=29.2568 df=2 P < 0.0001**

When the wing bones are broken down by individual bones the results are:

Coracoid - 4.2%

Humerus - 28.0%

Ulna - 28.7%

Carpometacarpus - 1.6

The radius sample (N=3) was too small to analyse.
